# Development and initial validation of the Chinese Version of the Noise Exposure Questionnaire (C-NEQ)

**DOI:** 10.1186/s12889-022-12648-5

**Published:** 2022-01-31

**Authors:** Kun Han, Qixuan Wang, Lu Yang, Sijia Xu, Chen Li, James Lin, Hao Wu, Zhiwu Huang

**Affiliations:** 1 Department of Otolaryngology-Head and Neck Surgery, Ninth People’s Hospital, Shanghai Jiao Tong University School of Medicine, Shanghai, China; 2 Ear Institute, Shanghai Jiao Tong University School of Medicine, Shanghai, China; 3 Shanghai Key Laboratory of Translational Medicine on Ear and Nose Diseases, Shanghai, China; 4 Network and Information Center, Shanghai Jiao Tong University, Shanghai, China

**Keywords:** Noise exposure questionnaire, Annual noise exposure, Reliability, Reproducibility, Chinese

## Abstract

**Background:**

With a high prevalence of noise-induced hearing loss (NIHL), the noise survey tools for identifying individuals with high risk of NIHL are still limited. This study was aimed to translate and develop a Chinese version of noise exposure questionnaire (C-NEQ), and validate its reliability and reproducibility.

**Methods:**

This study was conducted from May 2020 to March 2021 in China. The questionnaire was translated from the original NEQ and adapted into Chinese culture using the method according to the International Test Committee. Content validity was evaluated by our expert group. Construct validity and reliability of the C-NEQ was determined through estimating the confirmatory factor analysis and Cronbach’s alpha in a cross-sectional analysis among 641 Chinese speaking adults, respectively. The retest reproducibility of the C-NEQ was analyzed by using the intra-group correlation coefficient (ICC) in a follow-up analysis among 151 participants.

**Results:**

The C-NEQ comprises ten items covering four domains: occupational, housework, transport and recreational noise exposure. The annual noise exposure (ANE) was calculated as the protocol of original NEQ. A total of 641 adult participants (aged 26.9 ± 10.1 years, 53.4% males) completed the C-NEQ. The average time for completing the C-NEQ was 4.4 ± 3.0 min. Content validity indicated high relevance of the C-NEQ. The confirmatory factor analysis indices illustrated that the items of the C-NEQ were suitable with the data in the study. For the internal reliability, the Cronbach’s α coefficients of the total items and four domains (occupational, housework, transport, and recreational noise exposure) were 0.799, 0.959, 0.837, 0.825, and 0.803, respectively. Among them, 151 participants (aged 36.1 ± 11.1 years, 65.6% males) completed the retest of the C-NEQ 1 month after the first test. The ICC value of total ANEs between the first test and the second test was 0.911 (*P* < 0.001).

**Conclusions:**

In this study, we have validated the C-NEQ with adequate reliability and reproducibility for quantifying an individual’s annual daily noise exposure, which provides an effective fast-screen tool for researches and clinics to identify those individuals with high risks of NIHL within the short time duration (no more than five minutes) among Chinese population.

**Supplementary Information:**

The online version contains supplementary material available at 10.1186/s12889-022-12648-5.

## Introduction

According to the recently published inaugural World Report on Hearing (2021) [1], the World Health Organization (WHO) estimates that more than 1.5 billion people, or 20% of the world’s population, live with hearing loss. Nevertheless, the hearing loss can be prevented to some degree, especially the noise-induced hearing loss (NIHL) [2].

Noise exposure is the most common environmental factor causing the acquired hearing loss [3]. It is widely accepted that the risk of NIHL increases with long-term noise exposures above 80 dB, which increases significantly as the exposure rises above 85 dB [4]. It should be noted that not only the industrial or occupational noise exposures, but also the daily noise exposures (such as recreational and public transport noise exposure) may also cause the NIHL [5–7]. In a survey of the adolescent population in U.S., approximately three in four teenage students reported that they were once exposed to loud sound at school, and nearly half (46.5%) of students even reported that they were regularly exposed to loud sounds [8].

With regard to that over a billion people are at risk of avoidable hearing loss related to daily noise exposures globally [1], the accurate evaluation of individual noise exposure dose could help indicate one’s risk of NIHL and prevent from NIHL. However, it is challengeable to evaluate the long-tern noise exposure dose of each individual, and the effective self-reported tools used to quantify the daily noise exposure dose are still limited. In 2017, for quantifying both occupational and non-occupational noise exposure, Tiffany A. Johnson et al. developed the NEQ, which is a detailed, task-based questionnaire including 11 items for quantifying an individual’s annual noise exposure (ANE) [9]. The NEQ has been applied by a number of researchers among English speaking populations [10–12]. In a study among 106 gas-fired electric plant workers in Tanzania, researchers used the NEQ to obtain the self-reported ANE and used the noise logging dosimeter to measure the noise exposure level [12]. The results showed a significant correlation between the ANE score and measured noise exposure level, suggesting that the NEQ could be effective for evaluating the noise exposure level. However, to date, there are still no self-reported tools for evaluating daily noise exposure or application of the NEQ in China.

In this study, we aimed to translate the NEQ into a Chinese version, and adapt it to the Chinese culture relevance. For the first time, we applied this subjective questionnaire to quantify the individual noise exposure dose among Chinese population, and assess its validity and reproducibility.

## Methods

### Participants and study design

In this study, we recruited participants in China from May 2020 to March 2021. The inclusion criteria for the participants were (1) aged over 18 years; (2) able to read and understand Chinese; (3) committed to complete the questionnaire carefully. All the study procedures were carried out in accordance with relevant guidelines and approved by the ethics committee of the Ninth People’s Hospital affiliated to Shanghai Jiao Tong University School of Medicine. All the participants signed written informed consent forms.

Participants who completed the Chinese version of NEQ (C-NEQ) were included in the cross-sectional analysis for reliability. Part of participants were asked to complete the retest of the C-NEQ about 1 month after the first test. The responses to the retest were compared with those to the first test to assess the reproducibility.

### Translation and cultural adaptation of the NEQ

The original NEQ was developed by Tiffany A. Johnson et al. to quantify the individual annual noise exposure, including the occupational and daily exposure [9]. With the informed permission from Dr. Johnson’s team, we conducted the translation process using the method according to the International Test Committee [13]. First, two bilingual audiological technicians independently translated the original English questionnaire into Chinese. Then our bilingual expert team (including one professor of NIHL, two otological doctors and two audiological technicians) compared two versions of the translation, and a preliminary version of the C-NEQ was created after the consensus was reached. Second, the preliminary version of the C-NEQ was translated back into English by two independent English majors without knowledge of the original NEQ, then our expert team compared the back-translation with the original NEQ to ensure its’ meanings were consistent. Third, our expert team revised the differences in several items to make a further version of the C-NEQ for adapting it to the Chinese culture relevance and evaluate the content validity. Finally, a pilot study of the further version of the C-NEQ was conducted face-to-face with 10 Chinese speaking adults to evaluate their understanding of the questionnaire to develop the final version of the C-NEQ. The back-translation of the final C-NEQ has been confirmed by the developers.

### Statistical analysis

Data analysis was performed by using IBM SPSS 24.0 and AMOS 20.0software (SPSS Inc., Chicago, IL, USA). Continuous variables are expressed as the mean ± standard deviation (SD), and categorical variables are presented as percentages (n [%]). The content validity of the C-NEQ was evaluated by our expert team including five experts using the four-point scale from 1 (not relevant) to 4 (highly relevant), to assess the content effectiveness index (I-CVI) and scale content effectiveness index (S-CVI) as previously [14]. Items with I-CVI =1 and S-CVI values > 0.8 were accepted as indicating relevant content. Since the original NEQ is a practiced questionnaire, we used the confirmatory factor analysis (CFA) to determine the construct validity of the C-NEQ model based on 10 items [15]. Assessment indexes included the root mean square error of approximation (RMSEA < 0.1, acceptable), chi-squared values divided by the degrees of freedom (χ^2^/df < 3, acceptable), the goodness-of-fit index (GFI > 0.9, acceptable), normed fit index (NFI > 0.9, acceptable), non-normed fit index (NNFI > 0.9, acceptable), and comparative fit index (CFI > 0.9, acceptable). The minimum sample size in this study was calculated according to the requirement for CFA [16]. Cronbach’s α was calculated to analyze the internal consistency of the C-NEQ among all participants, values of which greater than or equal to 0.7 indicate satisfactory reliability [17]. The intra-group correlation coefficient (ICC > 0.7, high correlation) was performed to analyze the reproducibility among participants who retest the C-NEQ. A 2-tailed *P* < 0.05 was considered statistically significant.

## Results

### Culture adaptation and development of the C-NEQ

In comparison with the original English NEQ, two items were deleted: (1) How often were you around or did you shoot firearms such as rifles, pistols, shotguns, etc.; (2) Over the summer months, did you work a noisy paid job, such as in construction, farming, a factory, lawn service, carwash, or other indoor or outdoor job working around loud equipment or machinery. Besides, one item was added into the C-NEQ: How often do you take the public transportation such as subway and ferry (Q6). The other nine items and scales in the C-NEQ were similarly translated from the original NEQ.

Eventually, the C-NEQ comprises ten items covering four domains (Table 1, and full-text in the supplement): occupational noise exposure (Q1), housework noise exposure (Q2–4), transport noise exposure (Q5 and Q6) and recreational noise exposure (Q7–10), which required about 5 min to complete. The answer for each item consists of three aspects of self-evaluation during the past year: (1) the frequency or existence of the specified noise exposure (to answer ‘how many weeks a year’ for occupational exposure, to select ‘never, every few months, monthly, weekly, or daily’ for other exposure); (2) the duration of each time the noise exposure last (to answer ‘how many hours a week’ for occupational exposure, to select ‘≥ 8 hours, 4 to 8 hours, 1 to 4 hours, or<1 hour’ for other exposure); (3) The use of hearing protective devices.

**Table 1:** Items and calculation values of the C-NEQ

Domains	Items	Noise exposure activity	L (dBA)	Calculated T
Occupational				
	Q1	Occupational noise	**90**	**690**
Housework				
	Q2	Power tools	**94**	**274**
	Q3	Equipment/Machinery	**97**	**137**
	Q4	Motorized vehicles	**98**	**109**
Transport				
	Q5	Aircraft	**91**	**548**
	Q6	Subway/Ferry	**80**	**6953**
Recreational				
	Q7	Sporting/Entertainment	**94**	**274**
	Q8	Musical instrument	**87**	**1380**
	Q9	Music listening (earphones)	**76**	**17,520**
	Q10	Music listening (other speakers)	**78**	**11,037**
Outside items in C-NEQ	/	Routine sound exposures	**64**	**280,320**

### Calculation of the ANE by C-NEQ

Protocols used to calculate the ANE for each participant were referred to the original NEQ described previously [9], with partial modification for adapting the deleted and added items in the C-NEQ. In brief, the ANE was calculated as following:

The ‘C’ was assigned as the number of hours reported by the participant for the item/activity in the C-NEQ, which was calculated as following:

$$\mathrm{C}=\mathrm{frequency}\times \mathrm{duration}$$

The ‘C’ for Q1 can be calculated directly by ‘how many weeks a year’ and ‘how many hours a week’. For other items, were assigned the frequency values as follows: ‘never’ = 0, ‘every few months’ = 1, ‘monthly’ = 12, ‘weekly’ = 50, and ‘daily’ = 200; the duration values were as follows: ‘<1 h’ = 1, ‘1 to 4 hours’ = 3, ‘4 to 8 hours’ = 6, and ‘≥ 8 hours’=8.

The ‘T’ was assigned as the number of hours at which the item/activity is considered hazardous over a one-year time period. The ‘T’ was calculated as following:

$$\mathrm{T}=\frac{8760}{2^{\left(\frac{L-79}{3}\right)}}$$

Here the ‘L’ was assigned as the representative intensity of sound exposure of the activity according to previous studies [9, 18] (Table 1).

Besides, the daily routine sound exposures besides those above noise exposure activities should be taken into consideration, the ‘C_R_’ was calculated as 8760 minus the total ‘C’, and the ‘L_R_’ was about 64 dBA (Table 1).

The ‘D’ for the item/activity or routine sound exposures are calculated as following:

$$\mathrm{D}=\frac{\mathrm{C}}{\mathrm{T}}\times 100$$

Finally, the computations of ANE included 10 item/activity and the routine exposure dose as following:

$$ANE=\left[10\times {\mathit{\log}}_{10}\left(\frac{total\ D}{100}\right)\right]+79$$

### Study participants

In total, 641 participants aged 26.9 ± 10.1 years (range from 18 to 66) completed the C-NEQ. Among them, 342 (53.4%) were males, 258 (40.2%) reported the noise-exposed job. The details regarding the number, age, sex, and education of the participants are summarized in Table 2. For all participants, it took the mean time of 4.4 ± 3.0 min (range from 3.35 to 20.6) to complete the C-NEQ. The averaged ANE was 76.2 ± 7.4 dBA (range from 63.9 to 91.8).

**Table 2:** Characteristics of participants

Characteristics	Total (***n*** = 641)	Retest (***n*** = 151)
Age (year), mean (SD)	26.9 (10.1)	36.3 (11.1)
Sex, n (%)		
Male	342 (53.4)	99 (65.6)
Female	299 (46.6)	52 (34.4)
Education, n (%)		
Junior high school education or less	41 (6.4)	28 (18.5)
High school or junior college education	327 (51.0)	76 (50.4)
Bachelor degree	227 (35.4)	37 (24.5)
Master degree or above	46 (7.2)	10 (6.6)
Occupational noise exposure, n (%)		
Yes	258 (40.2)	95 (62.9)
No	383 (40.2)	56 (37.1)

### Content validity

The results of content validity analysis showed that the I-CVI of all items are 1, and the S-CVI of all items are higher than 0.8. That is, all experts indicated that the item contents were highly relevant, and at least four out of five experts indicated that the scale contents were highly relevant with the C-NEQ.

### Construct validity

To determine the construct validity of the C-NEQ, we performed factor model verification on the 10-item questionnaire. The results and relevant verification standard are shown in Table 3, suggesting that the model fulfilled the goodness-of-fit indices of CFA.

**Table 3:** Confirmatory factor analysis for the C-NEQ

Index	Value	Reference standard
χ^2^/df	2.066	< 3
GFI	0.937	> 0.9
RMSEA	0.069	< 0.1
CFI	0.929	> 0.9
NFI	0.908	> 0.9
NNFI	0.915	> 0.9

### Reliability

The reliability was confirmed for the total items and each domain of the C-NEQ by using the Cronbach’s α (Table 4). In this regard, Cronbach’s α of the occupational noise exposure, housework noise exposure, transport noise exposure, recreational noise exposure were estimated at 0.959, 0.837, 0.825, and 0.803, respectively. Cronbach’s alpha of the total items was further calculated at 0.799, thereby confirming the internal reliability of the C-NEQ.

**Table 4:** Cronbach’s α coefficient for the C-NEQ

Items	Cronbach’s α coefficient
Total	0.799
Occupational noise exposure	0.959
Housework noise exposure	0.837
Transport noise exposure	0.825
Recreational noise exposure	0.803

### Reproducibility

A total of 151 participants aged 36.1 ± 11.1 years (range from 20 to 63) completed a second test of the C-NEQ after a month of their first test. Among them, 99 (65.6%) were males, 95 (62.9%) reported the noise-exposed job. The averaged ANE of the first test was 77.6 ± 7.6 dBA (range from 64.0 to 91.5), and the averaged ANE of the second test was 77.1 ± 7.7 dBA (range from 64.0 to 88.4). The calculated ANEs of the first test was high correlated to ANEs of the second test (0.911, *P* < 0.001). ICC values of the 10 items scores ranged from 0.741 to 0.970, which was satisfactory. The ICC analysis indicated the adequate reproducibility of C-NEQ over a month (Table 5).

**Table 5:** The ICC analysis for both total ANE socres and the ten items scores

Items	ICC values	***P*** values
Total	0.911	< 0.001
Q1	0.937	< 0.001
Q2	0.753	< 0.001
Q3	0.854	< 0.001
Q4	0.790	< 0.001
Q5	0.838	< 0.001
Q6	0.845	< 0.001
Q7	0.741	< 0.001
Q8	0.970	< 0.001
Q9	0.862	< 0.001
Q10	0.759	< 0.001

## Discussion

In this study, by translating and adapting the original NEQ into Chinese culture, we developed the Chinese version of NEQ. For the first time, we validated the reliability and reproducibility of C-NEQ in 641 and 151 Chinese speaking adults, respectively. Our results suggested that the C-NEQ is potential to be a convenient and reliable self-reported tool for evaluating the daily noise exposure among Chinese population.

Regard with the increased prevalence of NIHL, the World Health Organization has recommended that the level of 75–80 dBA is the maximum permissible sound level for an 8-h time period to protect hearing [1, 4]. Recent studies have demonstrated that even a short-term noise exposure at moderate intensity could result in permanent damage in the cochlea [19, 20]. However, it is still difficult for individuals to clearly know that if they are under the hazard dose of noise exposure, since many sound exposures that were considered safe can actually be dangerous [21]. For instance, the study of transport noise exposure in Toronto suggested that the average noise level in the subway and bus reached 79.8 ± 4.0 dBA [22]. A large population follow-up study (*n* = 12,115) from the Trøndelag Health Study suggested that individuals with prolonged listening to a high sound volume of personal music players were associated with worse hearing thresholds (1.4 dB [0.1 to 2.8]) [23].

In general, the main methods to evaluate the noise exposure dose included using the sound monitoring equipment and questionnaire survey [24]. Although noise survey using the sound monitoring equipment is objective and accurate in a specific noise exposure environment during a period of time, it is time consuming and costly for evaluating the long-term noise exposure of each individual [25]. Therefore, the sound monitoring equipment is predominately applied in population with high levels of noise exposure such as industrial workers, construction workers, and miners [26–30].

The questionnaire survey is a subjective method to collect the self-reported data. In comparison with the sound monitoring equipment, questionnaire survey is more convenient and personalized for evaluating one’s daily noise exposure individually [17, 31]. To date, the questionnaire surveys used for evaluating the noise exposure are still limited. Lewkowski et al. have developed a questionnaire-based algorithm to assess the individual occupational noise exposure according to the worker’s cumulative task-based noise exposure levels [25]. Although Lewkowski et al.’s questionnaire might be useful in identification of workers with harmful occupational noise exposure, it’s not acceptable for assessment the comprehensive noise exposure including occupational and other daily noise. In this study, we chose to translate the Noise Exposure Questionnaire (NEQ) developed by Tiffany A. Johnson et al., because the NEQ was able to quantify the individual annual noise exposure (ANE) dose including occupational and non-occupational noises quickly (about 10 min) [9]. Although Hannah Guest et al. further developed a more detailed noise exposure structured interview for the estimation of lifetime noise exposure, it takes longer time (about half an hour) than the NEQ for most respondents [31].

Besides the translation, we have adapted the original NEQ into a Chinese version. Similarly with the original NEQ, the C-NEQ comprises assessment of general daily noise exposures arising from occupational, housework, transport and recreational noise sources. Particularly, two items were deleted from the original NEQ to simplify the C-NEQ, since those items are unnecessary according to Chinese culture. One item was about the use of shoot firearms such as rifles, pistols, shotguns, which is prohibited by law in China rather than western countries. The other item was about the occupational noise exposure during last summer, which was mainly designed for students. However, Chinese students usually have few opportunities to work the noise-exposed summer jobs in comparison with students from western countries. Thus, the item about the annual occupational noise exposure (Q1 in the C-NEQ) was enough for assessment in Chinese population. In addition, we added an item about the noise exposure during the public transportation such as subway and ferry. A number of studies have reported the noise exposure associated with various forms of public transportation, ranged from about 70 to 90 dBA [31–33]. Since China is a densely inhabited country with highly developed public transport networks, it is necessary to consider the noise exposure from daily transport for each individual, especially in cities.

According to the initial validation of the C-NEQ in this study, our results showed the reliability of total items and each domain (the Cronbach’s α >0.7) among 641 Chinese speaking adults. For the reproducibility of C-NEQ, the retested ANE scores were significantly correlated to the first ANE scores which were tested a month ago (the Pearson correlation coefficient = 0.911, *P* < 0.001). In general, here we have validated the adequate reliability and reproducibility of the C-NEQ. In addition, the averaged time for participants in this study to complete the C-NEQ was 4.4 ± 3.0 min, suggesting that the C-NEQ has the potential to be a fast-screen tool for quantifying the general daily noise exposure and for identifying those individuals who are at high risk for NIHL.

### Strengths and limitations

The main strength of our study is that it provides the first validated Chinese version of questionnaire to quantify the daily noise exposure, to our knowledge. By using the C-NEQ, we calculated the ANE scores of 641 Chinese adults (range from 63.9 to 91.8 dBA), which provides an indicator for identifying those high-risk individuals of NIHL. There are also limitations. First, we did not analyze the correlation between ANE scores and measured noise exposure dose, since the measured annual noise exposure levels of all participants were unavailable in this study. Second, we did not analyze the correlation between individual’s ANE score and hearing level, which could be further validated in future studies.

## Conclusions

According to the results of the study, we have validated the C-NEQ with adequate reliability and reproducibility for quantifying an individual’s general daily noise exposure in the past year, which provides a fast-screen tool for researches and clinics to identify those individuals with high risks of NIHL within the short time duration (no more than five minutes) among Chinese population.

## Supplementary Information

**Additional file 1.**

## Data Availability

All data generated or analyzed during this study are included in this manuscript**.** The full text of the C-NEQ is available from the corresponding author on reasonable request.

## References

[1] Chadha S, Kamenov K, Cieza A (2021). The world report on hearing, 2021. Bull World Health Organ.

[2] Wilson BS, Tucci DL, Merson MH, O'Donoghue GM (2017). Global hearing health care: new findings and perspectives. Lancet.

[3] Basner M, Babisch W, Davis A, Brink M, Clark C, Janssen S, Stansfeld S (2014). Auditory and non-auditory effects of noise on health. Lancet.

[4] Mirza R, Kirchner DB, Dobie RA, Crawford J (2018). Occupational noise-induced hearing loss. J Occup Environ Med.

[5] Wang Q, Qian M, Yang L, Shi J, Hong Y, Han K, et al. Audiometric phenotypes of noise-induced hearing loss by data-driven cluster analysis and their relevant characteristics. Front med (Lausanne). 2021;8(331). 10.3389/fmed.2021.662045 PMC8027076 33842516

[6] Neitzel RL, Fligor BJ (2019). Risk of noise-induced hearing loss due to recreational sound: review and recommendations. J Acoust Soc Am.

[7] Muller R, Schneider J (2017). Noise exposure and auditory thresholds of German airline pilots: a cross-sectional study. BMJ Open.

[8] Eichwald J, Scinicariello F (2020). Survey of teen noise exposure and efforts to protect hearing at school - United States, 2020. MMWR Morb Mortal Wkly Rep.

[9] Johnson TA, Cooper S, Stamper GC, Chertoff M (2017). Noise exposure questionnaire: a tool for quantifying annual noise exposure. J Am Acad Audiol.

[10] Ridley CL, Kopun JG, Neely ST, Gorga MP, Rasetshwane DM (2018). Using thresholds in noise to identify hidden hearing loss in humans. Ear Hear.

[11] Grinn SK, Wiseman KB, Baker JA, Le Prell CG (2017). Hidden hearing loss? No effect of common recreational noise exposure on Cochlear nerve response amplitude in humans. Front Neurosci.

[12] John W, Sakwari G, Mamuya SH (2018). Noise exposure and self-reported hearing impairment among gas-fired Electric Plant Workers in Tanzania. Ann Glob Health.

[13] Muñiz J, Elosua P, Hambleton RK (2013). International test commission guidelines for test translation and adaptation: second edition. Psicothema.

[14] Wang Y, Krska J, Lin B, Mei Y, Katusiime B, Guo Y, Zhang Z (2020). Cross-cultural adaptation and reliability testing of Chinese version of the living with medicines questionnaire in elderly patients with chronic diseases. Patient Prefer Adherence.

[15] Batista-Foguet JM, Coenders G, Alonso J (2004). Confirmatory factor analysis. Its role on the validation of health related questionnaires. Med Clin (Barc).

[16] Wolf EJ, Harrington KM, Clark SL, Miller MW (2013). Sample size requirements for structural equation models: an evaluation of power, Bias, and solution propriety. Educ Psychol Meas.

[17] McNeish D (2018). Thanks coefficient alpha, we'll take it from here. Psychol Methods.

[18] Neitzel R, Seixas N, Goldman B, Daniell W (2004). Contributions of non-occupational activities to total noise exposure of construction workers. Ann Occup Hyg.

[19] Qian M, Wang Q, Wang Z, Ma Q, Wang X, Han K, Wu H, Huang Z (2021). Dose-dependent pattern of Cochlear synaptic degeneration in C57BL/6J mice induced by repeated noise exposure. Neural Plast.

[20] Kujawa SG, Liberman MC (2009). Adding insult to injury: cochlear nerve degeneration after "temporary" noise-induced hearing loss. J Neurosci.

[21] Wang Q, Yang L, Qian M, Hong Y, Wang X, Huang Z, Wu H (2021). Acute recreational noise-induced Cochlear synaptic dysfunction in humans with Normal hearing: a prospective cohort study. Front Neurosci.

[22] Yao C, Ma AK, Cushing SL, Lin VYW (2017). Noise exposure while commuting in Toronto - a study of personal and public transportation in Toronto. J Otolaryngol Head Neck Surg.

[23] Engdahl B, Aarhus L (2021). Personal music players and hearing loss: the HUNT cohort study. Trends Hear.

[24] Sayler SK, Roberts BJ, Manning MA, Sun K, Neitzel RL (2019). Patterns and trends in OSHA occupational noise exposure measurements from 1979 to 2013. Occup Environ Med.

[25] Lewkowski K, McCausland K, Heyworth JS, Li IW, Williams W, Fritschi L (2018). Questionnaire-based algorithm for assessing occupational noise exposure of construction workers. Occup Environ Med.

[26] Huang FJ, Hsieh CJ, Young CH, Chung SH, Tseng CC, Yiin LM (2018). The assessment of exposure to occupational noise and hearing loss for stoneworkers in Taiwan. Noise Health.

[27] Neitzel RL, Svensson EB, Sayler SK, Ann-Christin J (2014). A comparison of occupational and nonoccupational noise exposures in Sweden. Noise Health.

[28] Ottoni AO, Barbosa-Branco A, Boger ME, Garavelli SL (2012). Study of the noise spectrum on high frequency thresholds in workers exposed to noise. Braz J Otorhinolaryngol.

[29] Sayler SK, Rabinowitz PM, Galusha D, Sun K, Neitzel RL (2019). Hearing protector attenuation and noise exposure among metal manufacturing workers. Ear Hear.

[30] Wang Q, Wang X, Yang L, Han K, Huang Z, Wu H (2021). Sex differences in noise-induced hearing loss: a cross-sectional study in China. Biol Sex Differ.

[31] Guest H, Dewey RS, Plack CJ, Couth S, Prendergast G, Bakay W, Hall DA (2018). The noise exposure structured interview (NESI): an instrument for the comprehensive estimation of lifetime noise exposure. Trends Hear.

[32] Neitzel R, Gershon RR, Zeltser M, Canton A, Akram M (2009). Noise levels associated with new York City's mass transit systems. Am J Public Health.

[33] Lee D, Kim G, Han W (2017). Analysis of Subway interior noise at peak commuter time. J Audiol Otol.

