# Supplementary material for: Development and initial validation of the Chinese Version of the Noise Exposure Questionnaire (C-NEQ)
Source: BMC Public Health. 2022 Jan 31;22:207. doi: 10.1186/s12889-022-12648-5 (PMC8805437; doi:10.1186/s12889-022-12648-5)
Supplement: Supplementary file 1 — Additional file 1. [file 12889_2022_12648_MOESM1_ESM.docx]

Supplement: Back-translation into English of the C-NEQ

| **Chinese version of Noise Exposure Questionnaire**  **INSTRUCTIONS:** Please answer the following questions about yourself, your hearing, and any noise you may have been around during the past year. Write an answer in the blank ______or check [ √ ] the best answer to each question. | | | | | |
| --- | --- | --- | --- | --- | --- |
| **Table 1. Occupational Noise Exposure (DURING THE PAST YEAR/12 MONTHS)** | | | | |  |
| **Q1. Did you work a noisy paid job, such as in construction, farming, a factory, lawn service, carwash, or other indoor or outdoor job working around loud equipment or machinery? By noisy job, we mean sounds so loud that you had to shout or speak in a raised voice to be heard at arm’s length.** | | | A. Yes (*If yes, please describe this noisy job: ___________________*)  B. No (*if no, skip to # Table 2*) |  |  |
| If you worked a noisy job, please estimate the number of weeks you worked in a year. There are 52 weeks in a year. | | | ________ Weeks |  |  |
| If you worked a noisy job, please estimate the number of averaged hours you worked in a week. | | | ________ hours worked per week |  |  |
| If you worked a noisy job, did you wear earplugs or earmuffs at work? | | | A. Never B. Sometimes C. Always |  |  |
| **Table 2. Non-occupational Noise Exposure (DURING THE PAST YEAR /12 MONTHS)** | | | | |  |
| Q2 | **Outside of a paid job, how often did you use power tools, chainsaws, or other shop tools?** | A. Never *(if never，skip to #Q3)*  B. Every few months  C. Monthly  D. Weekly  E. Daily | | |  |
|  | If you used power tools, on average, how many hours did each time/session last? | A. 8 hours or more  B. 4 hours up to 8 hours  C. 1 hour up to 4 hours  D. Less than 1 hour | | |  |
|  | If you used power tools, how often did you wear earplugs or earmuffs during this activity? | A. Never B. Sometimes C. Always | | |  |
| Q3 | **Outside of a paid job, how often did you drive heavy equipment or use loud machinery (such as tractors, trucks, or farming or lawn equipment like mowers/leaf blowers)?** | A. Never *(if never，skip to #Q4)*  B. Every few months  C. Monthly  D. Weekly  E. Daily | | |  |
|  | If you drove/used loud machinery, on average, how many hours did each time/session last? | A. 8 hours or more  B. 4 hours up to 8 hours  C. 1 hour up to 4 hours  D. Less than 1 hour | | |  |
|  | If you drove/used machinery, how often did you wear earplugs or earmuffs during this activity? | A. Never B. Sometimes C. Always | | |  |
| Q4 | **How often did you ride/operate motorized vehicles such as motorcycles, jet skis, speed boats, snowmobiles, or four-wheelers?** | A. Never *(if never，skip to #Q5)*  B. Every few months  C. Monthly  D. Weekly  E. Daily | | |  |
|  | If you rode motorized vehicles, on average, how many hours did each time/session last? | A. 8 hours or more  B. 4 hours up to 8 hours  C. 1 hour up to 4 hours  D. Less than 1 hour | | |  |
|  | If you rode motorized vehicles, how often did you wear earplugs or earmuffs during this activity? | A. Never B. Sometimes C. Always | | |  |
| Q5 | **How often did you ride in or pilot small aircraft/private airplanes?** | A. Never *(if never，skip to #Q6)*  B. Every few months  C. Monthly  D. Weekly  E. Daily | | |  |
|  | If you flew airplanes, on average, how many hours did each time/session last? | A. 8 hours or more  B. 4 hours up to 8 hours  C. 1 hour up to 4 hours  D. Less than 1 hour | | |  |
|  | If you flew airplanes, how often did you wear earplugs or earmuffs during this activity? | A. Never B. Sometimes C. Always | | |  |
| Q6 | **How often do you take the public transportation such as subway and ferry?** | A. Never *(if never，skip to #Q7)*  B. Every few months  C. Monthly  D. Weekly  E. Daily | | |  |
|  | If you take the public transportation, on average, how many hours did each time/session last? | A. 8 hours or more  B. 4 hours up to 8 hours  C. 1 hour up to 4 hours  D. Less than 1 hour | | |  |
|  | If you take the public transportation, how often did you wear earplugs or earmuffs during this activity? | A. Never B. Sometimes C. Always | | |  |
| Q7 | **How often did you attend the events with amplified public announcement/music systems such as car/truck races, commercial/high school sporting events, music concerts, dances, or KTV, etc.?** | A. Never *(if never，skip to #Q8)*  B. Every few months  C. Monthly  D. Weekly  E. Daily | | |  |
|  | If you attended these events, on average, how many hours did each time/session last? | A. 8 hours or more  B. 4 hours up to 8 hours  C. 1 hour up to 4 hours  D. Less than 1 hour | | |  |
|  | If you attended these events, how often did you wear earplugs or earmuffs during this activity? | A. Never B. Sometimes C. Always | | |  |
| Q8 | **How often did you play a musical instrument?** | A. Never *(if never，skip to #Q9)*  B. Every few months  C. Monthly  D. Weekly  E. Daily | | |  |
|  | If you played, please tell us what musical instrument. |  | | |  |
|  | If you played a musical instrument, on average, how many hours did each time/session last? | A. 8 hours or more  B. 4 hours up to 8 hours  C. 1 hour up to 4 hours  D. Less than 1 hour | | |  |
|  | If you played a musical instrument, how often did you wear earplugs or earmuffs while playing? | A. Never B. Sometimes C. Always | | |  |
| Q9 | **How often did you listen to music, radio programs, etc. using personal headsets or earphones?** | A. Never *(if never，skip to #Q10)*  B. Every few months  C. Monthly  D. Weekly  E. Daily | | |  |
|  | If you listened through earphones, on average, how many hours did each time/session last? | A. 8 hours or more  B. 4 hours up to 8 hours  C. 1 hour up to 4 hours  D. Less than 1 hour | | |  |
| Q10 | **Other than music concerts and headset use, how often did you listen to music, radio programs, etc. from audio speakers in a car or at home?** | A. Never *(if never，you’re done with the survey)*  B. Every few months  C. Monthly  D. Weekly  E. Daily | | |  |
|  | If you listened via speakers, on average, how many hours did each time/session last? | A. 8 hours or more  B. 4 hours up to 8 hours  C. 1 hour up to 4 hours  D. Less than 1 hour | | |  |
